# Adjunctive Treatment Effect of Non-Thermal Atmospheric Pressure Plasma in Periodontitis-Induced Rats

**DOI:** 10.3390/jcm14030896

**Published:** 2025-01-29

**Authors:** Hee-Young Choi, Hyun-Joo Kim, Ju-Youn Lee, Ji-Young Joo

**Affiliations:** 1 Department of Periodontology, Dental and Life Science Institute, School of Dentistry, Pusan National University, Yangsan 50612, Republic of Korea; 202397513@pusan.ac.kr (H.-Y.C.); periohjkim@pusan.ac.kr (H.-J.K.); heroine@pusan.ac.kr (J.-Y.L.); 2 Department of Periodontology, Dental Research Institute, Pusan National University Dental Hospital, Yangsan 50612, Republic of Korea

**Keywords:** alveolar bone loss, cold plasma, non-thermal atmospheric pressure plasma, periodontal debridement, periodontal diseases, periodontitis

## Abstract

**Background/Objectives**: As non-thermal atmospheric pressure plasma (NTP) is known to have advantages in application in the medical field, we consider its applicability to periodontitis, a representative chronic inflammatory disease. The purpose of this study was to evaluate the effect of NTP in inhibiting the progression of periodontitis in a rat model when additionally used in scaling and root planing (SRP). **Methods**: To induce experimental periodontitis in 20 rats, ligatures were placed in the maxillary second molar and lipopolysaccharide from *Porphyromonas gingivalis* was injected around the teeth. Then, NTP treatment was performed for 2 or 5 min, together with scaling and root planing (SRP). To evaluate alveolar bone loss, micro-computed tomography (micro-CT) analysis and hematoxylin–eosin (H-E) staining were performed. Tartrate-resistant acid phosphatase (TRAP) analysis was performed to compare the number of osteoclasts, while immunohistochemistry (IHC) analysis was performed to determine the expression levels of receptor activator of nuclear factor-*κ*B ligand (RANKL) and osteoprotegerin (OPG). Enzyme-linked immunosorbent assay (ELISA) analysis was performed for the detection of cytokines (TNF-α, IL-1β, and IL-10) in tissues and sera. **Results**: When SRP was combined with NTP, alveolar bone loss was decreased, the number of osteoclasts and RANKL expression were decreased, OPG expression was increased, and pro-inflammatory cytokine (TNF-α and IL-1β) levels were significantly decreased. Compared with the NTP treatment for 2 min, when treated for 5 min, less alveolar bone loss, fewer osteoclasts, a lower RANKL expression level, and a higher OPG expression level were observed. **Conclusions**: This study evaluated the adjunctive treatment effect of NTP in periodontitis-induced rats. Based on the results of this study, we suggest that supplemental NTP treatment may be a good option for non-surgical periodontal treatment; however, further studies are needed to elucidate the mechanism through which NTP suppresses periodontal inflammation.

## 1. Introduction

Periodontitis is a chronic inflammatory disease in which periodontal tissue is lost due to the secretion of inflammatory cytokines and matrix metalloproteinase as a result of pathological changes in the subgingival environment, which is caused by an inflammatory response of the gingiva due to bacterial plaque deposition [1]. The inflammatory response of the gingiva can be expressed differently depending on the host’s systemic condition. Therefore, determining whether periodontitis develops and progresses does not seem to be a specific bacterial or putative toxic factor, but, rather, a host inflammation and immune response to the bacterial complex [2,3,4]. That is, disease is not caused by individual pathogens, but rather by a disruption of periodontal tissue homeostasis associated with an ecologically balanced biofilm due to polymicrobial synergy and imbalance [5].

The periodontal microflora normally maintains homeostasis in relation to the host; however, when pathologic shifts occur, dysbiosis of the subgingival microflora can occur. Therefore, periodontal treatment needs to be approached from this point of view [4,6]. Depending on the level of periodontal inflammation, the heterogeneity of the angiogenic process varies, which can be determined by the microvessel density (MVD). Increased MVD can lead to higher exposure of various cytokines, adhesion molecules, and other inflammatory factors. Modulating this vessel flow may be helpful in the treatment of periodontitis [7]. In all periodontal diseases, the first step in the treatment process is directed toward motivation, targeting behavioral changes to achieve adequate self-performed oral hygiene practices and controlling local and systemic modifiable risk factors (e.g., smoking). The second step is focused on professional interventions to reduce and possibly eliminate supra- and subgingival biofilms and calculus. The main component of the second step is mechanical instrumentation combined with effective self-performed plaque control measures, leading to shifts in the subgingival ecology and host responses. Disruption of the microbial biofilm, alterations in the microbial composition, and suppression of the inflammatory tissue infiltrate may be implemented as components of the second step of non-surgical periodontal treatment [8]. SRP is the gold standard of treatment in terms of mechanical debridement, but is not always effective in changing the composition of the subgingival microbiota or improving clinical parameters for initially deep pockets or areas that are anatomically difficult to access [9]. Therefore, in some cases, adjunctive therapy, such as laser treatment [10] or chemotherapy [11], may be required. Although these adjuvant treatments can improve clinical outcomes, they are not optimal due to their limited positive effects or side effects [12,13,14].

Non-thermal atmospheric pressure plasma (NTP)—also called cold atmospheric pressure plasma—is a partly ionized form of plasma which can be generated by applying energy to a gas that is not directly biologically effective (e.g., argon, helium, and nitrogen) at low or atmospheric pressure [15]. Through continuous research on NTP, NTP devices have been developed and applied to the medical field based on multi-disciplinary global research [15,16,17]. As it uses a low temperature, NTP does not inflict thermal injuries on the tissue. In addition, various effects have been demonstrated: inactivation of a wide range of microorganisms, including multi-drug-resistant bacteria [18,19]; stimulation of cell proliferation and tissue regeneration [17,18,19,20]; and potential for application in oncology due to the inactivation of cells through initializing programmed cell apoptosis under high-intensity plasma treatment [21,22]. NTP has been shown to present pro-apoptotic effects more efficiently in tumor cells than in normal cells through altering the redox balance in such a way that oxidative distress leads to cell death in the context of head and neck cancer [23]. A recent systematic review has demonstrated that NTP is a promising method for root canal disinfection in endodontic therapy [24].

According to recent studies applying NTP in the field of dentistry, NTP has been shown to have significant effects on teeth whitening [25], fluoride retention on the enamel surface [26], and eradication of pathogenic bacteria [27]. Nevertheless, few studies have been conducted in the periodontic field. In this study, an NTP-generating machine using argon (Ar) gas was devised. The purpose of this study was to evaluate whether the additional application of NTP to SRP for the treatment of periodontitis could inhibit the progression of periodontitis in a periodontitis-induced rat model through measuring the amount of alveolar bone loss, number of osteoclasts, expression levels of RANKL and OPG, and inflammatory cytokine secretion.

## 2. Materials and Methods

### 2.1. Animals

A total of 20 7-week-old female Sprague Dawley rats (average body weight: 180–200 g) were included in the experiment. To allow the rats to adapt to the laboratory environment, 6-week-old rats were obtained from a trading company (Hana trading Co., Pusan, Republic of Korea) to ensure a 1-week adaptation period before the experiment. Rats were housed at 18–25 °C under standard conditions with food and water provided ad libitum in the Experimental Animal Center of Pusan National University. All the experimental protocols and treatment procedures were approved by the Institutional Animal Care and Use Committee of Pusan National University (PNU-2019-2385).

### 2.2. Experimental Design

The sample size was determined considering a power of 80% and *p*-value of 0.05, which was calculated using the G*power software (v3.1.9.4; Universität Kiel, Kiel, Germany) [28]. The 20 rats were randomly divided into the following 5 groups, with 4 rats per group: an untreated control group (C group) and 4 ligature-induced periodontal disease groups, namely, a ligation-only group (L group), a ligation and SRP treatment group (LR group), a ligation and SRP followed by NTP treatment for 2 min group (LRP2 group), and a ligation and SRP followed by NTP treatment for 5 min group (LRP5 group). All procedures were performed after induction of anesthesia using intraperitoneal injection of ketamine hydrochloride (Yuhan Ketamine 50 Inj^®^., Yuhan Co., Seoul, Republic of Korea; 100 mg/kg body weight) and xylazine hydrochloride (Rompun^®^, Bayer Co., Leverkusen, Germany; 10 mg/kg body weight).

**Figure 1:**
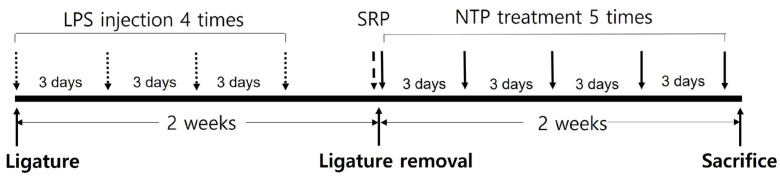
Flowchart showing the experimental design. LPS injection and NTP treatment were performed once every 3 days. LPS: lipopolysaccharide; SRP: scaling and root planing; NTP: non-thermal atmospheric pressure plasma.

To induce experimental periodontitis in the treatment groups (i.e., L, LR, LRP2, and LRP5), 5-0 silk ligatures (Ailee Co., Pusan, Republic of Korea) were placed around the cemento-enamel junction (CEJ) of the maxillary left second molar subgingivally and maintained for 2 weeks. During this period, 10 µL of PBS solution containing 1 mg/mL lipopolysaccharide from *Porphyromonas gingivalis* (InvivoGen, San Diego, CA, USA) was injected into the gingival tissue around the teeth once every 3 days. Two weeks later, the ligatures were removed and SRP and NTP treatments were started. SRP was performed once on the day of removal of ligatures only in the LR, LRP2, and LRP5 groups using a hand instrument (11/12 Mini Five™, Hu-Friedy Co., Chicago, IL, USA). NTP treatment was applied around the teeth of animals in the LRP2 and LRP5 groups for 2 min and 5 min, respectively. This was performed using a scanning method once every 3 days for 2 weeks with an NTP-generating device (Feagle Co., Yangsan, Republic of Korea). The NTP treatment time was set referring to the conditions described in previous studies [26,27,28,29]. At 2 weeks after NTP treatment, all rats were sacrificed using carbon dioxide asphyxiation (Figure 1).

### 2.3. Micro-CT Analysis

Micro-CT analysis of the maxillary block sections was performed with micro-computed tomography (SkyScan1173, Bruker-CT, Kontich, Belgium) at a resolution of 8.88 ㎛ (90 kVp for tube voltage, 88 μA for tube current). The acquired raw data were reconstructed using the NRecon software (v1.6.8.0; Bruker-CT). The cross-sectional images were aligned with an axis using a Dataviewer (v1.5.1.2; Bruker-CT). As an analysis program, Ct Analyzer (v1.14.4.1; Bruker-CT) was used. The region of interest (ROI) was defined as the volume of alveolar bone between the first and second molar. The ROI was selected using a slice-based method starting, with the first slice containing the CEJ of the first molars and moving apically for 57 slices. To determine the alveolar bone loss, the bone volume per total volume (BV/TV) was calculated.

### 2.4. Histomorphometric Analysis

After specimens were fixed with buffered neutral formalin (Sigma Aldrich, St. Louis, MO, USA) for 1 week, decalcification was performed for about 4 weeks using 10% EDTA solution (pH 7.2). Decalcified samples were dehydrated by increasing the ethanol concentration. Subsequently, after undergoing a clearing process with xylene, they were embedded in paraffin (Leica Biosystems Richmond Inc., Richmond, IL, USA) to obtain blocks. Then, blocks were sliced to 4 μm using an Automated Rotary Microtome (Leica RM2255, Leica Biosystems, Wetzlar, Germany). Deparaffinization and hydration were performed for further staining.

For hematoxylin–eosin (H-E) staining, Mayer’s hematoxylin (Dako™, Dako North America Inc., Carpinteria, CA, USA) and 1% eosin Y reagent (BBC Biochemical, Vernon, WA, USA) were used, and, after ethanol dehydration, ethanol was removed with xylene. For tartrate-resistant acid phosphatase (TRAP) staining, sections were stained with TRAP staining solution (0.1 mg/mL of naphthol AS-MX phosphate, 0.3 mg/mL of Fast Red Violet LB stain; Sigma Aldrich) according to the manufacturer’s instructions. After staining and mounting, images were obtained using a microscope/digital slide scanner (Pannoramic™ 250 Flash III, 3DHISTECH Co., Budapest, Hungary).

In H-E staining images, the distance from the CEJ of the first and second molar to the alveolar bone crest (ABC) was measured to determine the alveolar bone loss. In TRAP staining images, the number of osteoclasts per square millimeter found in the alveolar bone between the first and second molar was measured.

### 2.5. Immunohistochemistry Analysis of RANKL and OPG Expression

Before immunohistochemistry (IHC) staining, deparaffinization, hydration, and washing were performed. Heat-induced epitope retrieval was performed using citrate-based buffer (pH 6.0) in a cooker for 10 min at 95 °C. For blocking of endogenous peroxidase activity, 3% H_2_O_2_ was used. For blocking of proteins to prevent non-specific antibody binding, bovine serum albumin was used.

For antibody incubation, the primary antibodies used to observe receptor activator of nuclear factor-κB ligand (RANKL) and osteoprotegerin (OPG) expression were rabbit anti-RANKL antibody (PA5-21951, 1:800, Thermo Fisher Scientific, Waltham, MA, USA) and rabbit anti-OPG antibody (ab203061, 1:800, Abcam, Cambridge, UK), respectively. Diluted primary antibodies were pipetted onto the sections and incubated for one hour at 37 °C. Then, diluted secondary antibodies were added for one hour at 37 °C. The secondary antibody used was Discovery Universal secondary antibody (760-4205, Ventana Medial Systems Inc., Oro Valley, AZ, USA), and these antibodies were directly conjugated to horseradish peroxidase.

After the secondary antibody was washed off, 3,3′-diaminobenzidine was pipetted onto the sections. A counterstain was performed with hematoxylin (Ventana Medial Systems Inc., Oro Valley, AZ, USA), followed by alcohol dehydration and clearing with xylene. These IHC staining procedures were performed using an IHC research instrument (Discovery Ultra™, Ventana Medical Systems Inc.), according to the manufacturer’s instructions. Then, the mounting process was performed to observe the slide under the microscope.

From the images obtained using the microscope/digital slide scanner (3DHISTECH Co.) after staining, the ImageJ software (NIH image, Bethesda, MD, USA) was used to calculate the stained area of the ROI. The ROI was defined as the periodontal tissue between the maxillary first and second molar teeth.

### 2.6. Inflammatory Cytokine Evaluation in Periodontal Tissue and Serum

Immediately after sacrificing the rats, gingival tissue near the first and second molars and blood was obtained. Serum was separated from the blood. For the evaluation of pro-inflammatory cytokines such as tumor necrosis factor-α (TNF-α), interleukin-1β (IL-1β), and anti-inflammatory cytokine interleukin-10 (IL-10), a colorimetric sandwich enzyme-linked immunosorbent assay (ELISA) kit (Abcam) was used according to the manufacturer’s instructions, and measurement was performed with a microplate reader (Synergy HTX plate reader, Biotek, Winooski, VT, USA).

### 2.7. Statistical Analysis

Statistical analysis was performed using SPSS (v24.0; IBM Corp., Armonk, NY, USA). The values of demographic features are presented as the mean ± standard deviation (SD). The normality test was performed using the Shapiro–Wilk test. For the results of micro-CT and TRAP staining analyses, comparative analysis among groups was performed using the Kruskal–Wallis H test followed by the post hoc Bonferroni test. For the analysis of other results, comparative analysis among groups was performed using one-way analysis of variance (ANOVA), followed by the post hoc Scheffé test. Statistical significance was accepted at a level of *p* < 0.05.

## 3. Results

### 3.1. Micro-CT Analysis for Alveolar Bone Resorption Evaluation

**Figure 2:**
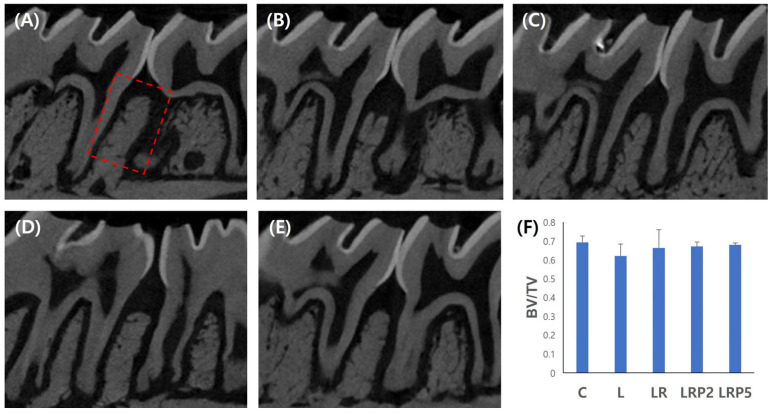
Effect of NTP on alveolar bone resorption. Representative micro-CT images of (**A**) C group, (**B**) L group, (**C**) LR group, (**D**) LRP2 group, and (**E**) LRP5 group; (**F**) bar graph showing bone volume per total volume for the 5 groups. Red dashed box: ROI. NTP: non-thermal atmospheric pressure plasma. BV/TV: bone volume/total volume. Data represent mean ± SD (N = 4/group). No statistically significant differences among groups.

To determine the effect of NTP on alveolar bone resorption, micro-CT imaging was performed to obtain BV/TV values. A high BV/TV value indicates less alveolar bone loss, and it can be considered that alveolar bone repair is promoted. The lowest BV/TV value was observed in the L group (0.62 ± 0.02). Compared to the L group, the LR (0.65 ± 0.03), LRP2 (0.66 ± 0.01), and LRP5 (0.67 ± 0.00) groups showed higher BV/TV values. However, there were no statistically significant differences among the groups (Figure 2).

### 3.2. Histomorphometric Analysis and Quantification of Osteoclasts

**Figure 3:**
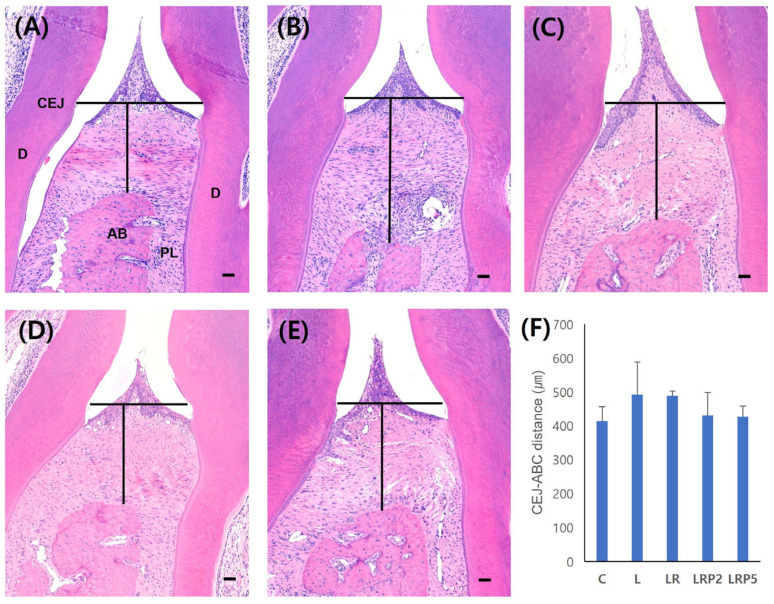
Effect of NTP on alveolar bone resorption. Representative H-E staining images of (**A**) C group, (**B**) L group, (**C**) LR group, (**D**) LRP2 group, and (**E**) LRP5 group; (**F**) distance between CEJ and ABC. NTP: non-thermal atmospheric pressure plasma; PL: periodontal ligament; D: dentin; AB: alveolar bone; CEJ: cemento-enamel junction; ABC: alveolar bone crest. Scale bars = 50 μm. Data represent mean ± SD (N = 4/group). No statistically significant differences among groups.

H-E staining was performed to measure the CEJ-ABC distance (μm). The shorter the distance, the less the alveolar bone loss was. In the L group, the largest distance value (491.7 ± 96.8), elongation of the epithelium, infiltration of inflammatory cells, and alveolar bone loss were observed. Compared to the L group, the LR (488.2 ± 12.5), LRP2 (430.1 ± 67.9), and LRP5 (426.2 ± 31.8) groups showed shorter distances. In addition, reductions in inflammatory cell infiltration and less alveolar bone loss were observed. However, there were no statistically significant differences among the groups (Figure 3).

**Figure 4:**
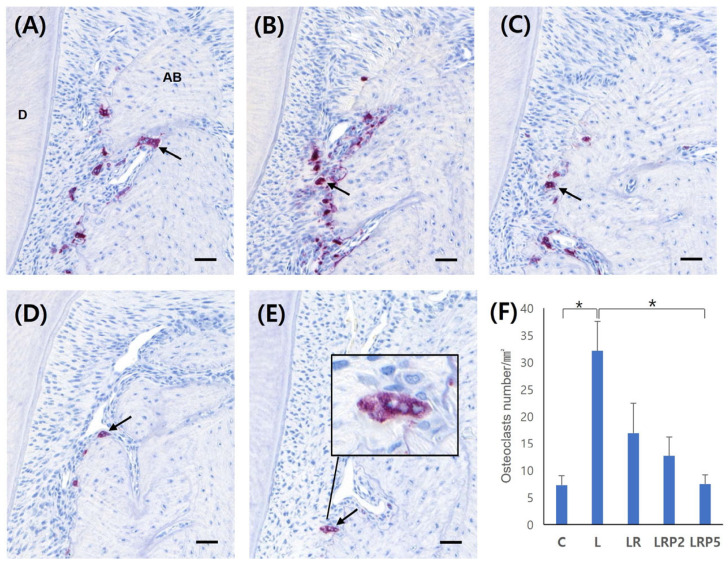
Effect of NTP on osteoclast formation. Representative TRAP staining images of (**A**) C group, (**B**) L group, (**C**) LR group, (**D**) LRP2 group, and (**E**) LRP5 group; (**F**) quantification of osteoclast number. NTP: non-thermal atmospheric pressure plasma; AB: alveolar bone; D: dentin; black arrows: multinucleated osteoclasts. Scale bars = 50 μm. Data represent mean ± SD (N = 4/group). *: statistically significant difference (*p* < 0.01).

TRAP staining was performed to measure the number of osteoclasts identified in the alveolar bone between the first and second molar. The smaller the number of osteoclasts, the less bone destruction occurred. The largest number of osteoclasts was observed in the L group (32.08 ± 6.61). Compared to the L group, the LR (16.88 ± 6.60), LRP2 (12.70 ± 4.11), and LRP5 (7.50 ± 2.00) groups showed fewer osteoclasts. In particular, when compared to the L group, significantly small amounts of osteoclasts were observed in the LRP5 group (*p* < 0.01) (Figure 4).

### 3.3. Immunohistochemistry Analysis of RANKL and OPG Expression

**Figure 5:**
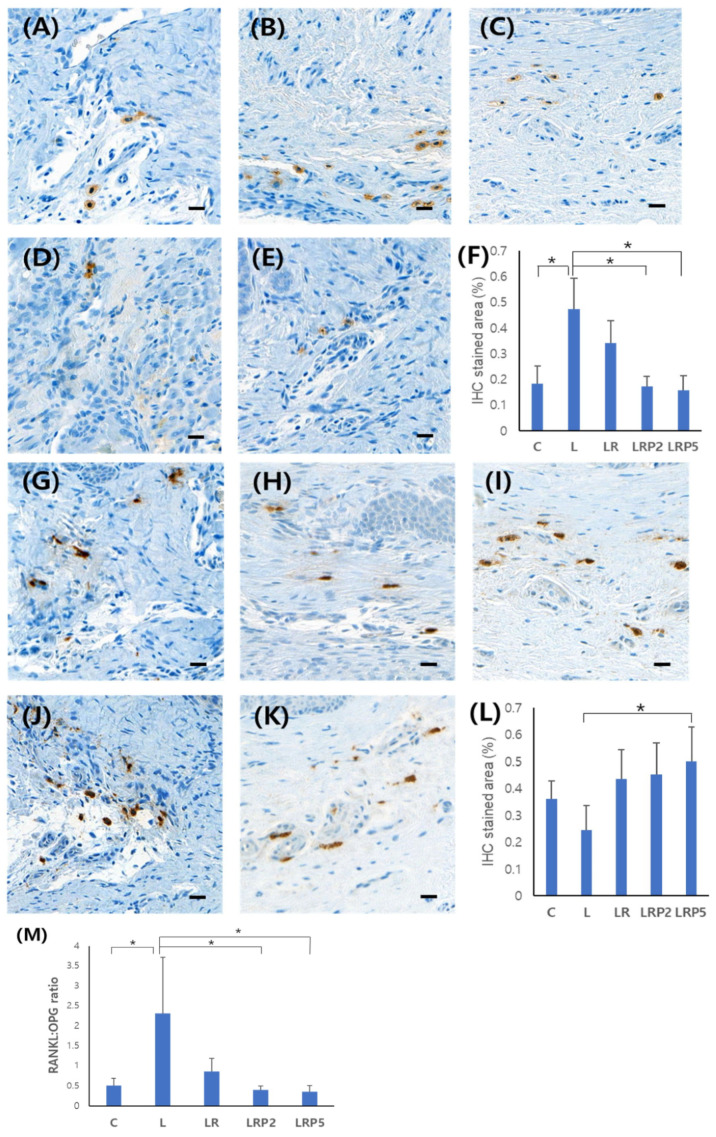
Effect of NTP on the expression of RANKL and OPG. Representative IHC analysis images of (**A**) C group, (**B**) L group, (**C**) LR group, (**D**) LRP2 group, and (**E**) LRP5 group on RANKL expression; (**F**) IHC-stained area (%) on RANKL expression; IHC analysis images of (**G**) C group, (**H**) L group, (**I**) LR group, (**J**) LRP2 group, and (**K**) LRP5 group on OPG expression; (**L**) IHC-stained area (%); (**M**) RANKL/OPG ratio. NTP: non-thermal atmospheric pressure plasma. Scale bars = 20 μm. Data represent mean ± SD (N = 4/group). *: statistically significant difference (*p* < 0.05).

The expression amount was compared by calculating the percentage of IHC-stained areas in the images. RANKL showed a statistically significantly higher expression level in the L group (0.47 ± 0.12). In particular, the LRP2 (0.17 ± 0.03) and LRP5 (0.15 ± 0.25) groups showed significantly lower expression levels (*p* < 0.01) (Figure 5A–F). In the case of OPG, a high expression level was shown in the treatment groups compared to the low level in the L group (0.24 ± 0.12). In particular, the LRP5 group (0.50 ± 0.11) showed a significantly higher expression level (*p* < 0.05) (Figure 5G–L).

The RANKL/OPG ratio was significantly higher in the L group (2.31 ± 1.40) and significantly lower in the LRP2 (0.39 ± 0.19) and LRP5 (0.35 ± 0.20) groups (*p* < 0.05) (Figure 5M).

### 3.4. Inflammatory Cytokine Evaluation in Periodontal Tissue and Serum

**Figure 6:**
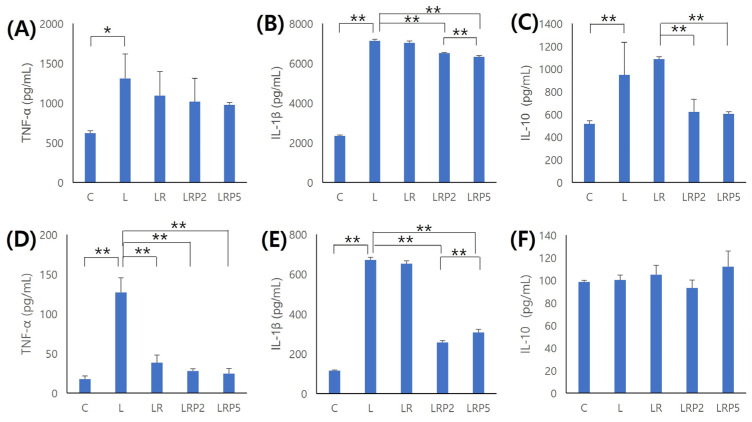
Effect of NTP on the expression of inflammatory/immune-related cytokines. (**A**–**C**) Detection amount of cytokine from tissue; (**D**–**F**) detection amount of cytokine from serum. (**A**,**D**) TNF-α, (**B**,**E**) IL-1β, and (**C**,**F**) IL-10. NTP: non-thermal atmospheric pressure plasma. Data represent mean ± SD (N = 4/group). *: statistically significant difference (*p* < 0.05). **: statistically significant difference (*p* < 0.01).

The amount of TNF-α detected in tissue was significantly higher in the L group than the C group, and tended to decrease in the LR, LRP2, and LRP5 groups (Figure 6A). Moreover, TNF-α detected in serum was significantly higher in the L group than the C group, and significantly lower in the LR, LRP2, and LRP5 groups (*p* < 0.01) (Figure 6D). IL-1β detected in tissue was significantly higher in the L group than the C group, and significantly lower in the LRP2 and LRP5 groups. In particular, compared to the LRP2 group, the LRP5 group showed significantly lower values (*p* < 0.01) (Figure 6B). IL-1β detected in serum was similar to the result in tissue (*p* < 0.01) (Figure 6E). IL-10 detected in tissue was significantly higher in the L group than the C group, and significantly lower in the LRP2 and LRP5 groups than the LR group (*p* < 0.01) (Figure 6C). IL-10 detected in serum was higher in the LR and LRP5 groups than L group, but the difference was not statistically significant (Figure 6F).

## 4. Discussion

Species and combinations of anaerobic Gram-negative bacteria, including *Aggregatibacter actinomycetemcomitans, Porphyromonas gingivalis, Prevotella intermedia, Treponema denticola,* and *Tannerella forsythia*, are strongly implicated in the pathogenesis of periodontitis, and their complete eradication is very difficult [30]. Mechanical debridement including SRP with oral hygiene, according to professional instruction, is considered a typical method for controlling the supra- and subgingival bacterial biofilm and calculus in the early treatment of periodontitis. The clinical endpoint of periodontal treatment may be defined as no bleeding on pocket probing and pocket closure; that is, a probing pocket depth of ≤4 mm. Pocket reduction or “pocket closure” as an outcome variable has been validated by data showing a lower risk for disease progression and, eventually, tooth loss for shallow remaining sites [8,9]. However, there are limitations, such as limited access of the instrument due to tooth or anatomical factors [31,32], as well as difficulty in removing periodontal pathogens that have infiltrated periodontal tissues [33]. Adjunctive therapies have been developed to not only overcome these limitations, but also compensate for reduced effectiveness due to impaired host defense mechanisms [14]. Thus, the following supplementary treatments have been used: laser therapy, which can induce hemostasis and better wound healing [34]; photodynamic therapy, which can increase the bactericidal effect of the laser [35]; host modulation therapies, which alter the destructive aspect of the host response; and chemotherapeutic therapy, which can be applied topically or systemically and may also reduce the self-destructive immune response of the host to pathogens [36,37].

Various useful effects of NTP have been reported, and its development is currently underway for use in the field of dentistry [16,38]. The effects of NTP are due to the generation of reactive oxygen species (ROS), which are known to play a dominant role in plasma-induced biological reactions. The ROS affect immune cells, promote the proliferation of keratinocytes, and exhibit antimicrobial effects. NTP developed based on these characteristics has shown potential as an adjunctive therapy, in addition to the advantages of other adjunctive therapies described above. Its hemostatic effect, promotion of wound healing, sterilization effect, the fact that it does not injure tissue due to its low-temperature characteristics, and ease of access to periodontal tissue due to the gas form support the potential of this approach [15,16,17]. In addition, these characteristics of NTP can have positive effects on maintaining the homeostasis of the periodontal microbiota.

When the effect of NTP on alveolar bone loss was evaluated, less alveolar bone loss was observed in both micro-CT and histomorphometric analyses. This was also consistent with the results of a previous study [39], but there were no statistically significant differences among the groups. However, we confirmed that additional NTP treatment inhibited osteoclast formation.

RANKL and OPG are both cytokines that bind to the receptor activator of nuclear factor-*κ*B (RANK) and have opposite effects. It has been reported that higher RANKL and lower OPG levels are observed in areas where periodontal destruction is active, compared to those with healthy periodontal conditions [40]. Moreover, the RANKL/OPG ratio in gingival tissue is higher in periodontitis than in healthy tissue [41,42], and the ratio decreases after periodontal treatment [43]. This is consistent with the present experimental results, suggesting the possibility that adjunctive NTP treatment induces inhibition of periodontitis progression by affecting the expression levels of RANKL and OPG involved in osteoclast differentiation. Subsequently, the altered balance between RANKL and OPG activity may affect bone resorption or bone formation.

In reference to a previous study in which serum samples from periodontitis patients showed high variability and low detection frequency of cytokine concentration [44], inflammatory tissues were also collected for cytokine analysis. As with reports of increased TNF-α and IL-1β levels in gingival tissue and serum from patients with periodontitis [44,45,46], the present study also showed higher levels of TNF-α and IL-1β in the L group. In addition, in the LR, LRP2, and LRP5 groups, lower levels of TNF-α and IL-1β were observed in both tissue and serum samples, similar to the finding that TNF-α and IL-1β levels were correlated with the degree of periodontitis and decreased after SRP [47,48,49]. This shows that periodontal treatment and the application of adjunctive NTP reduce the expression of TNF-α and IL-1β, thus inhibiting the stages in which osteoclasts are activated through RANK, and alveolar resorption occurs.

IL-10 is an anti-inflammatory cytokine that suppresses the production of pro-inflammatory cytokines and RANKL expression in activated T cells [50]. The results of this study showed a higher IL-10 value in the L group; however, the IL-10 levels in serum samples did not differ significantly among groups. The IL-10 level in tissue samples was significantly higher in the LR group, and showed significantly lower values in the LRP2 and LRP5 groups. These results could be interpreted in the same context as previous results indicating that IL-10 has dual roles—one of suppressing the secretion of cytokines (e.g., TNF-α and IL-1β) and an immunostimulatory function of stimulating B-cells—thus leading to bone resorption, and that the activity may vary depending on the local cytokine environment [51]. In addition, considering the process of feedback and homeostasis control between cytokines in the extensive cytokine network associated with periodontal disease, it could be regarded as reflecting an increase in anti-inflammatory mediators to counteract the pro-inflammatory mediators that rise in response to bacterial changes.

Dental plasma devices are being developed and can be set up with various working gases and treatment conditions; therefore, further studies under more subdivided conditions (type of working gas, NTP treatment time, and treatment frequency of NTP) are needed. In this study, through performing various analyses, it was confirmed that NTP affects the progression of periodontitis, and elevated pro-inflammatory cytokine levels control the expression of RANKL and OPG, thereby leading to osteoclast activation and alveolar bone loss. The present study confirmed that, when SRP and NTP treatments are combined, changes in the major factors involved in the progression of periodontal disease were observed and, as a result of these positive changes, the progression of periodontitis could be suppressed and alveolar bone loss could be reduced.

Therefore, we suggest that supplementary NTP treatment could be a good choice for non-surgical periodontal treatment. In addition, it is significant that the present study is the first in vivo study on the effect of supplementary NTP with different conditions in periodontal treatment. However, this study has limitations: it did not measure periodontal clinical indices such as PPD, CAL, BOP, and PI in the periodontal tissue of rats. Probing pocket depth is a critical clinical index for evaluating the outcome of periodontal treatment. However, there is currently no widely used device that can measure probing pocket depth in small units such as those used in rat experiments.

## 5. Conclusions

A combination of SRP and NTP treatment reduced the number of osteoclasts, pro-inflammatory cytokine production, and the expression level of RANKL while promoting the expression level of OPG in a periodontitis-induced rat model. The longer NTP treatment time increased its effectiveness; that is, it was more effective in inhibiting the progression of periodontitis. Although further research is needed on the detailed mechanism of NTP treatment and its effectiveness under various conditions, this study demonstrates the potential for adjunctive NTP treatment to be selected as a method to suppress the progression of periodontitis.

## Data Availability

All datasets generated and/or analyzed during the present study are available from the corresponding author on reasonable request.
